# Laparoscopic subtotal cholecystectomy for Mirizzi syndrome: A report of a case

**DOI:** 10.1016/j.ijscr.2019.01.010

**Published:** 2019-01-19

**Authors:** Jiro Kimura, Naokazu Takata, Alan Kawarai Lefor, Masaki Kanzaki, Ken Mizokami

**Affiliations:** a Department of Surgery, Tokyo Bay Urayasu Ichikawa Medical Center, Chiba, Japan; b Department of Surgery, Jichi Medical University, Tochigi, Japan

**Keywords:** CT, computed tomography, MRCP, magnetic resonance cholangiopancreatography, ERCP, endoscopic retrograde cholangiopancreatography, Laparoscopic subtotal cholecystectomy, Mirizzi syndrome, Cholelithiasis

## Abstract

**Introduction:**

Mirizzi syndrome is a rare complication of gallstone disease. The purpose of this report is to describe the utility of laparoscopic subtotal cholecystectomy for Mirizzi syndrome.

**Presentation of case:**

A 53-year-old female presented with dark urine and right upper quadrant pain. Blood tests revealed elevated liver and biliary enzyme levels. Magnetic resonance cholangiopancreatography showed a narrowed common hepatic duct compressed by a large gallstone, consistent with Mirizzi syndrome. Semi-urgent laparoscopic cholecystectomy was planned. At operation, circumferential dissection of the gallbladder neck was difficult. The fundus of the gallbladder was opened and a 2 cm stone extracted. The gallbladder neck was sutured and a drain placed. The postoperative clinical course was uneventful.

**Discussion:**

After laparoscopic cholecystectomy in patients with Mirizzi syndrome, complication rates, including bile duct injuries, is high. In patients with Mirizzi syndrome, removal of the responsible stone is the main purpose of treatment.

**Conclusion:**

Laparoscopic subtotal cholecystectomy is a useful technique for patients with Mirizzi syndrome to avoid bile duct injury.

## Highlights

• Laparoscopic subtotal cholecystectomy was successfully performed for a patient.
• The rates of bile duct injuries is high after laparoscopic cholecystectomy.
• Removal of the stone is the main purpose of treatment.
• Laparoscopic subtotal cholecystectomy is useful for patients with Mirizzi syndrome.

## 1 Introduction

Mirizzi syndrome is a rare complication of gallstone disease, with a reported incidence between 0.06% and 5.7% in patients undergoing cholecystectomy [1,2]. It was named after Pablo Mirizzi, who defined the syndrome as a benign common hepatic duct obstruction due to gallstone impaction in the gallbladder neck resulting in local inflammation and bile duct spasm [3]. Mirizzi syndrome was finally attributed to extrinsic compression of the common hepatic duct by gallstones impacted in the cystic duct or the gallbladder neck [4]. Bile duct wall necrosis and subsequent cholecysto-biliary fistula caused by chronic inflammation is a rare consequence of the disease. The purpose of the present report is to describe the utility of laparoscopic subtotal cholecystectomy for Mirizzi syndrome. This work has been reported in line with the SCARE criteria [5].

## 2 Presentation of a case

**Fig. 1:**
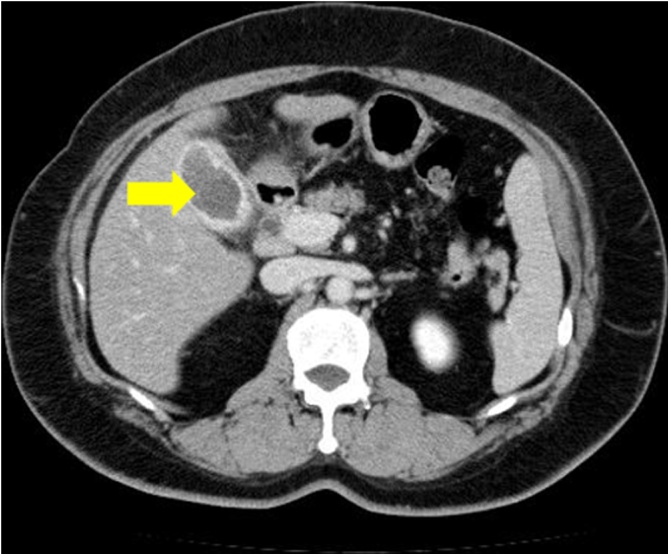
Abdominal computed tomography scan showed a large gallbladder stone (arrow) as a low-density area but no stones in the common bile duct.

**Fig. 2:**
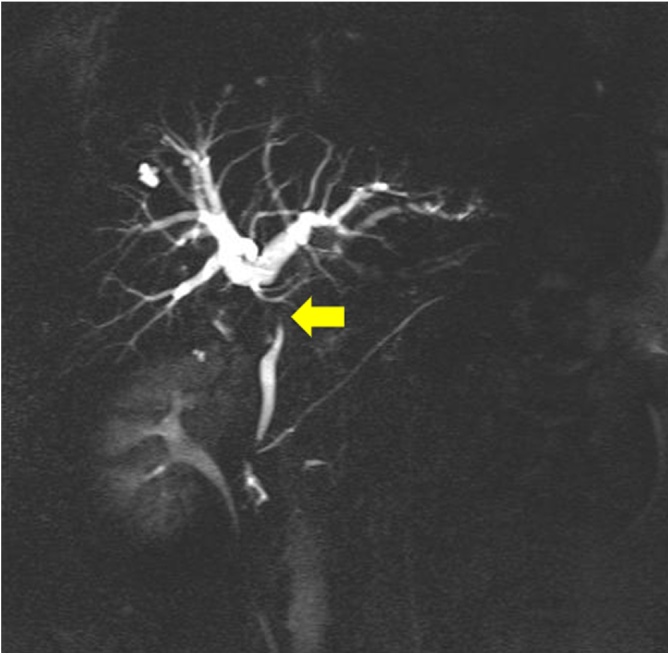
Magnetic resonance cholangiopancreatography showed a narrowed common hepatic duct (arrow) compressed by a large gallstone and dilated intrahepatic bile ducts.

**Fig. 3:**
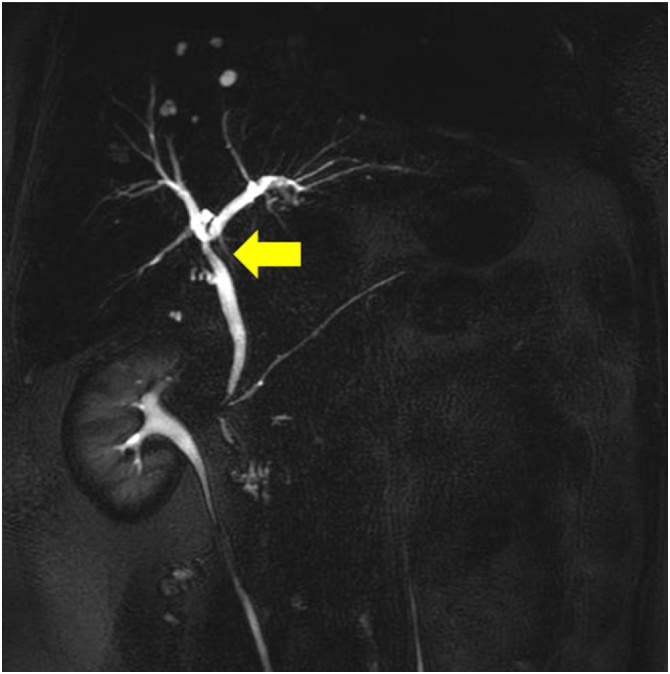
Follow-up magnetic resonance cholangiopancreatography one month later showed that the previously demonstrated (Fig. 2) narrowing of the common hepatic duct was resolved to normal caliber.

A 53-year-old female who previously underwent open hysterectomy for a fibroid tumor, presented to an outside facility with postprandial abdominal pain. She was noted to be jaundiced and laboratory studies revealed elevated biliary enzymes. She was referred for further examination. She had mild jaundice and mild right upper quadrant tenderness. Laboratory studies showed elevated liver and biliary enzymes (total bilirubin 5.5 mg/dl). Abdominal computed tomography (CT) scan showed a stone in the gallbladder but none in the common bile duct (Fig. 1). She was initially thought to have passed a stone through the common bile duct, and cholecystectomy was planned. However, one month later, she presented again with a one-week history of dark urine. She also described clay-colored stool and occasionally had right upper quadrant pain. Physical examination showed no abnormalities. Blood tests revealed elevated liver and biliary enzymes and recurrent common bile duct stones were suspected. Magnetic resonance cholangiopancreatography (MRCP) showed a narrowed common hepatic duct extrinsically compressed by a large gallstone and dilated intrahepatic bile ducts (Fig. 2), establishing the diagnosis of Mirizzi syndrome. Laparoscopic cholecystectomy was then performed semi-urgently. At operation, the duodenum was densely adherent to the body of the gallbladder, and the neck of the gallbladder could not be dissected circumferentially. The fundus of the gallbladder was opened and a 2 cm stone causing the extrinsic duct compression was removed. The neck of the gallbladder was sutured and a drain placed. The postoperative clinical course was uneventful and she was discharged. Follow-up MRCP performed one month postoperatively showed that the narrowed common hepatic duct had resolved to normal caliber (Fig. 3).

## 3 Discussion

The most widely accepted classification of Mirizzi syndrome was proposed by McSherry et al. [6], who described two types: Type I includes partial or complete obstruction of the common hepatic duct due to external compression and Type II refers to the formation of a communication between the gallbladder neck or the cystic duct and the common hepatic duct. Csendes et al. further subclassified cholecysto-biliary communication into three types according to the diameter of fistula [7]. In this classification, Type I lesions are those with external compression of the common bile duct, Type II is a cholecysto-biliary fistula that involves less than one-third of the circumference of the bile duct, Type III is a fistula that involves up to two-thirds of the bile duct circumference, and Type IV is a fistula with complete bile duct destruction. The patient in this report are classified as Type I.

Shortly after the advent of laparoscopy for the treatment of gallbladder disease, Rust et al. suggested that Mirizzi syndrome may be a contraindication for laparoscopic cholecystectomy [8]. In 1992, Paul et al. reported the first successful laparoscopic treatment of Type I Mirizzi syndrome [9], and several cases were described thereafter. This approach has not gained wide acceptance, partly because of the lack of large series due to the rarity of the syndrome and the controversial results in existing reports.

If the diagnosis of Mirizzi syndrome is made preoperatively, ERCP can be both diagnostic and therapeutic before surgery, as stenting across the obstruction allows decompression of the common bile duct [10]. For patients who are unsuitable surgical candidates, endoscopic retrograde cholangiopancreatography with stenting can be definitive treatment.

A systematic review of 10 case series reported that laparoscopic cholecystectomy was successful in 73/124 patients (59%). Patients in studies reporting a high rate of preoperatively establishing the diagnosis had a significantly lower risk for conversion (p < 0.05), procedure-related complications (p < 0.05), and reoperation (p < 0.05), when compared with studies of patients with a lower rate of preoperative diagnosis [11]. Therefore, preoperative diagnosis of Mirizzi syndrome is the key to successful laparoscopic treatment.

In rare cases, chronic inflammation may result in necrosis of the bile duct wall and erosion of the anterior or lateral wall of the common bile duct by impacted stones leading to cholecysto-biliary (cholecysto-hepatic or cholecysto-choledochal) fistulae [2]. Therefore, patients with Mirizzi syndrome should be treated early. However, the inflammatory reaction can lead to difficulty in identifying structures during surgery. After laparoscopic cholecystectomy in patients with Mirizzi syndrome, complication rates up to 60% and bile duct injuries in up to 22% were reported in the literature [12]. A few prior case series have demonstrated the feasibility of a laparoscopic approach to Mirizzi syndrome. However, there is a high conversion rate to open surgery of up to 30% and the techniques for reconstruction, if needed, are a challenge [13,14]. Therefore, laparoscopic subtotal cholecystectomy is recommended for such patients. Although there are some reports about laparoscopic subtotal cholecystectomy for Type I Mirizzi syndrome [[15], [16], [17]], none of them mentioned about details of management and tips of surgical techniques. Removal of the impacted stone, which is the culprit of this entity, is the important key point for successful management of Type I Mirizzi syndrome. This technique requires subtotal resection of wall of the gallbladder, removal of the impacted stone, and placement of a drain with or without closing the stump of the gallbladder. For difficult gallbladders accompanied by severe cholecystitis, bile duct injury is reported in only 0.08% [18]. Laparoscopic subtotal cholecystectomy is useful in patients with Mirizzi syndrome to avoid this complication.

## 4 Conclusion

In patients with Mirizzi syndrome, removal of the impacted stone is the main purpose of treatment. Laparoscopic subtotal cholecystectomy is useful in patients with Mirizzi syndrome to avoid bile duct injury.

## Conflicts of interest

All authors have no conflict of interest.

## Sources of funding

Authors had no sources of funding.

## Ethical approval

IRB/Ethics Committee ruled that approval was not required for this study.

## Consent

Written informed consent was obtained from the patients for publication of this case report and accompanying images. A copy of the written consent is available for review by the Editor-in-Chief of this journal on request.

## Author’s contribution

The work presented was carried out in collaboration between all authors. JK, AKL, NT, MK, and KM defined the research theme, discussed analyses and approved the final version to be published. JK analyzed the data, interpreted the results and wrote the paper.

## Registration of research studies

There is no need to register because it is a case report.

## Guarantor

Jiro Kimura.

## Provenance and peer review

Not commissioned, externally peer-reviewed.
